# Enhancing eXcellence Through Community, Empowerment, and Learning (EXCEL): a Pilot Study of a Self-Determination Theory-Informed Peer-Facilitated Psychoeducational Intervention for Health Sciences Trainees

**DOI:** 10.1007/s40670-026-02770-z

**Published:** 2026-06-06

**Authors:** S. N. Mladen, Y. Zhai, C. A. Gorman, D. A. Rogers, R. Rampe, S. McCarthy, D. Watson, L. M. Boitet

**Affiliations:** 1 Department of Physical Medicine and Rehabilitation, Virginia Commonwealth University (VCU) School of Medicine, Richmond, VA USA; 2 School of Human Development and Organizational Studies in Education, University of Florida, Gainesville, FL USA; 3 Department of Management, Information Systems, and Quantitative Methods, UAB, Birmingham, AL USA; 4 UAB Medicine Office of Wellness, Birmingham, AL USA; 5 Department of Surgery, UAB, Birmingham, AL USA; 6 Department of Psychiatry and Behavioral Neurobiology, UAB, Birmingham, AL USA; 7 Department of Human Studies, UAB, Birmingham, AL USA; 8 Department of Medical Education, UAB, Birmingham, AL USA

**Keywords:** Self-determination theory, Trainee, Peer support, Professional development

## Abstract

Health sciences trainees experience substantial distress from factors derived from the learning environment. Yet traditional training programs rarely provide structured opportunities to build autonomy-supportive nontechnical skills needed to navigate these challenges. To address this gap, we developed and evaluated a novel peer-led psychoeducational group intervention designed for health sciences trainees grounded in Self-Determination Theory (SDT). This program aimed to strengthen trainees’ access to resources supporting competence, relatedness, and autonomy through three structured components: psychoeducation, guided discussion, and targeted skill-development. A pre-post design was used to collect Work-Related Basic Psychological Need Satisfaction Scale (BPNS-W) and perceived stress data, and the post-session survey included items from the Session Wants and Needs Outcome Measure (SWAN-OM) and two general evaluation questions. Mean-level paired analyses demonstrated a significant increase in resource acquisition related to autonomy, and perceived stress decreased significantly from pre- to post-session. Importantly, latent change score modeling revealed substantial variability in individual trajectories, and indicated trainees with lower baseline basic psychological need satisfaction showed the largest gains, which were statistically significant across all three SDT domains. Trainees positively evaluated the program, reporting that they felt listened to, and were able to identify self-support strategies and explore their feelings. Participants also indicated that they found the sessions helpful and were likely to attend future sessions. Together, this study supports the utility of an SDT-informed, peer-facilitated intervention model for reducing trainee perceived stress and strengthening resources in support of basic psychological need satisfaction.

## Introduction

Health sciences training, specifically biomedical sciences, medicine, and health professions disciplines, has traditionally centered on building technical skills and conceptual knowledge. However, an expanding body of literature highlights that long-term success in health science careers also depends on the development of professional and career-related skills [1–3]. Despite their importance, such skills are seldom taught explicitly or systematically within health sciences training programs [4–8], leaving many trainees to navigate the psychosocial challenges of their training environments without the necessary skillsets. This gap is particularly concerning, given the high prevalence of distress and burnout among health sciences trainees [9, 10]. Together, these challenges highlight a need for upstream support mechanisms that proactively equip trainees with the skills required to navigate common challenges of training, rather than relying on downstream, reactive interventions when distress has already emerged.

Importantly, the learning environment plays a critical role in shaping trainee outcomes [11]. Prior research demonstrates that professional skill development is associated with improved self-efficacy, collaboration, and confidence [12], with downstream implications for innovation and work performance [13, 14]. Conversely, insufficient professional skills negatively impact motivation, retention, wellbeing, and career transitions [7, 15]. Positive professional experiences, effective mentorship, and supportive organizational culture have significant positive impacts on wellbeing, productivity, and retention [16–20]. Trainees who feel supported in their roles are more likely to thrive [21, 22], whereas those subjected to toxic or unresponsive academic environments are at higher risk of burnout and disengagement [23–26].

Despite the known influence of the learning environment on trainee outcomes, most interventions in health sciences education focus on individual-level factors, with comparatively limited attention to the broader training context [11]. Notably, prior work has identified that 60% of trainees in the health sciences experience distress [9], a threshold associated with an increased risk of burnout, turnover, and suicidal ideation [27–29]. A low sense of recognition, poor role clarity, increased moral distress, and heightened social isolation and loneliness, factors that are largely embedded within the training environment itself, were significantly associated with high distress scores [30]. These findings underscore a critical need for evidence-based approaches that are grounded in theoretical frameworks capable of guiding intervention design and that target the factors that underpin trainee wellbeing.

While peer-support and wellness initiatives have become increasingly common in health sciences education [31], their structures vary and many are not explicitly designed within a theoretical framework aligned with their intended outcomes. As a result, these interventions often emphasize general support and reflection without systematically targeting the drivers of distress derived from the learning environment. Further, many approaches focus on individual-level wellness strategies (e.g., mindfulness and self-care) that do not fully address the structural or contextual contributors to distress [11]. Recently, there has been a call for interventions that more directly target the learning environment and adopt autonomy-supportive principles [11]. While such shifts in faculty development and institutional culture are essential, a complementary gap remains in the skillsets of the learners themselves. Accordingly, autonomy-supportive approaches should extend at the level of learner development to build capacity for proactive engagement with common challenges in training.

### Theoretical Framework

The intervention described in this study addresses this need by introducing a structured, peer-facilitated professional development intervention explicitly designed using Self-Determination Theory (SDT) as an organizing framework. SDT posits that wellbeing and motivation are driven by fulfillment of the basic psychological needs of competence, autonomy, and relatedness [32, 33]. Briefly, competence is defined as feeling effective and capable in the work environment, including the sense of being able to learn, master challenges, and expand upon one’s abilities. Relatedness refers to the experience of mutual trust, belongingness with, and connection to others. Autonomy refers to an individual’s ability to act in ways that are reflective of their own values and interests, with a sense of volition rather than exclusively external pressure [34]. Interestingly, when these needs are supported, individuals are more likely to engage in adaptive behaviors such as proactively seeking and utilizing resources that support their functioning and growth [35]. In contrast, inadequate satisfaction, or frustration, of basic psychological needs negatively impacts trainee wellbeing and creates a critical gap in trainees’ learning experience [36–39]. Accordingly, SDT serves as a promising framework for guiding the design of interventions that aim to systematically support trainee wellbeing.

Peer-based approaches are particularly well-suited to support basic psychological need acquisition. Emerging evidence suggests peer support by way of helping others promotes a sense of community and agency [40], with benefits including self-management, empowerment, and the achievement of positive work habits and career goals [41]. As neuroscience-informed models, such as the Neurosequential Model (NM), postulate, positive peer support likely increases a trainee’s ability to achieve nervous system regulation and access safety within educational spaces to learn effectively [42]. The NM principle of sequential processing follows the pattern of regulate, relate, and reason from a bottom-up brain processing experience to engage with information. This suggests that learners cannot effectively engage in relational or cognitive learning processes in dysregulating or psychologically unsafe environments. Peer-based interventions may be particularly well-suited to supporting regulation and learning, as reduced power differentials can enhance perceptions of safety, agency, and connection, thereby enabling access to higher-order reasoning and skill acquisition [43], closely aligned with SDT. Integrating a clear theoretical framework within a peer-facilitated format may therefore represent an important advancement over existing models that emphasize relational support or skill development in isolation.

While SDT has been widely used in health sciences education [44–47], its use has primarily focused on explaining learner motivation with less emphasis on guiding the structured design of interventions [11]. Further, many existing peer-support and group-based interventions often neglect to consistently target specific psychological mechanisms. As a result, there remains a need for intervention models that more explicitly translate theoretical constructs into reproducible program design. In this context, SDT offers a useful framework for structuring interventions with hypothesized mechanisms of change. By centering competence, relatedness, and autonomy as core design targets, SDT provides a mechanism for intentionally aligning intervention components with desired outcomes. Accordingly, interventions that are explicitly designed to support the acquisition of these needs may be better positioned to produce consistent improvements in resource utilization and stress reduction compared to less structured approaches.

A novel aspect of this study therefore is the use of SDT as a design framework to explicitly structure intervention components around the targeted satisfaction of basic psychological needs. This represents a shift from general peer-support or wellness programming toward a mechanism-driven intervention model, in which each component is intentionally mapped to a specific psychological process associated with wellbeing. Further, the present study uses a collaborative partnership between the health sciences and counseling training programs, leveraging the expertise of advanced master’s-level counseling trainee clinicians as session facilitators. These facilitators occupy a unique, quasi-professional role, combining formal training in evidence-based counseling approaches with a peer-adjacent positionality relative to participants. This structure enables the delivery of psychologically informed, structured sessions while preserving the relatability and reduced power differential characteristic of peer-led interventions. In turn, counseling trainees gain supervised experience in group facilitation, while participants receive support from facilitators trained to foster belonging, agency, and reflective practice. This theory-based, dual-benefit model thus represents a novel and scalable approach to intervention delivery within health sciences training environments.

The present study aimed to evaluate the effectiveness, feasibility, and acceptability of a peer-facilitated, SDT-guided professional development intervention for health sciences trainees. The primary objective of this study was to evaluate whether participation in the intervention enhanced trainees’ perceptions of basic psychological need satisfaction and reduced perceived stress. We hypothesized that, compared to pre-intervention levels, trainees would report significantly higher autonomy-, competence- and relatedness-related resource acquisition following session participation. We further hypothesized that trainees would report significantly lower perceived stress post-intervention compared to pre-intervention. Our secondary objective was to assess the feasibility and acceptability of the intervention, with the goal of describing a scalable and adaptable model for implementation across health sciences training contexts.

## Materials and Methods

### Context

This study took place at a large academic medical center in the southeastern United States and included graduate and postgraduate trainees across health sciences units: Joint Health Sciences (biomedical sciences), the Medical School (medical students, physician-scientist students, and residents and fellows), the Nursing School, and the School of Health Professions (physician assistant, occupational therapy, and physical therapy students). Trainees were recruited using a convenience sampling method via monthly email communications to electively participate in monthly 60-min group sessions. Multiple communication channels were used, including targeted email distribution to students subscribed to institutional listservs, announcements in university newsletters, and redistribution by program managers.

**Fig. 1:**
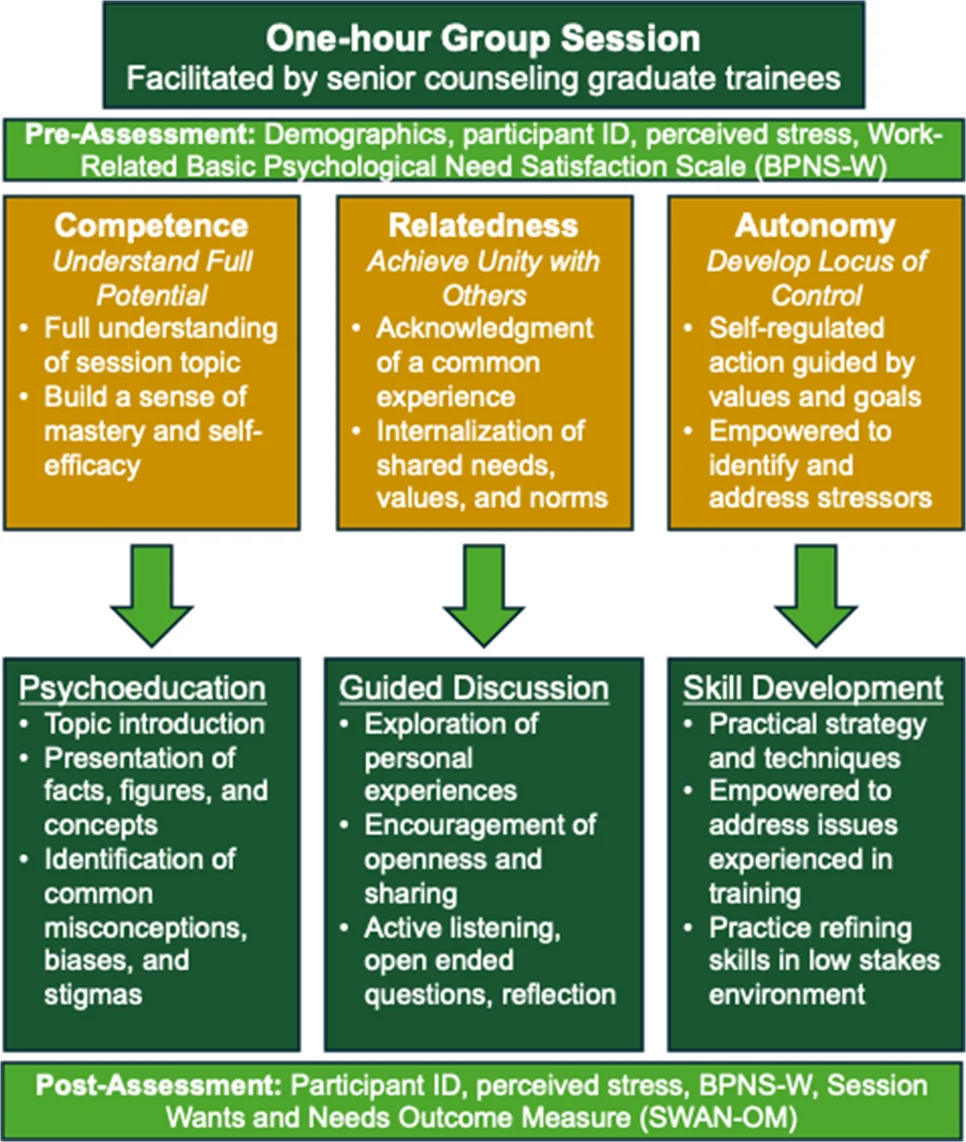
Conceptual framework for Enhancing eXcellence through Community, Empowerment, and Learning (EXCEL) intervention. Sessions are organized into three 20-minute components aligned with core SDT psychological needs. The Psychoeducation component supports competence by enhancing understanding of the session topic, clarifying key concepts and misconceptions, and fostering a sense of mastery. The Guided Discussion component addresses relatedness by facilitating shared reflection, normalizing common experiences, and encouraging open exploration of personal perspectives. The Skill Development component targets autonomy by teaching practical skills and strategies, and gives opportunities to practice application of new skills to in a safe environment. These components were designed to provide trainees with comprehensive resources to effectively manage common demands within their training environments

The intervention described is a 60-minute peer-facilitated professional development framework, with topics chosen from trainee suggestions and included the following: Work-life Balance, Autonomy, Identity Formation, Role Clarity, Adaptability, Anxiety, Cognitive Control, Interpersonal Relationships, Change Management, Managing Uncertainty, Boundaries, Wellbeing, and Solution-Focused Coping. Sessions were designed to support trainees’ basic psychological needs and reduce perceived stress. Each session was structured into three distinct sections in approximately 20-min blocks: Psychoeducation, Guided Discussion, and Skill Building, all mapped to the basic psychological needs posited by SDT (Fig. 1). The Psychoeducation section was designed to support the need for competence by enhancing trainees’ understanding of the session topic, clarifying key concepts and misconceptions, and fostering a sense of mastery. The Guided Discussion section addressed the need for relatedness by creating space for shared reflection, acknowledgment of common experiences, and open exploration of personal perspectives related to the topic. Finally, the Skill Development section targeted the need for autonomy by equipping trainees with practical strategies and techniques, allowing them to practice and apply new skills to proactively navigate challenges. This study evaluated participating trainees’ perceptions of basic psychological need satisfaction, perceived stress, and the feasibility and acceptability of the intervention. This study used data from 15 independently delivered 60-minute sessions, each evaluated using a pre- and post-session assessment completed by participating trainees. The study protocol was reviewed and approved by the study institution’s institutional review board (IRB-300012190).

Sessions were intentionally “by trainees, for trainees,” absent of faculty, staff, or administrators to maintain relatability and reduce the power differential between session facilitators and participants. Each session was led by two senior-level trainee clinicians enrolled in the Master of Arts in Counseling program at the study institution. Trainee clinicians were considered “peers” because, like participants, they shared trainee status and were navigating training programs with rigorous requirements and demands. This is consistent with the literature’s definition of peer as “*people from similar social groupings who are not professional teachers helping each other to learn and learning themselves by teaching*” [48] Senior-level clinicians were in the final year of their training and actively providing clinical services in the institution’s Community Counseling Clinic, giving them both the experience and credibility to facilitate sessions while remaining relatable to participants. In total, 10 trainee clinicians served as peer facilitators across the intervention.

Facilitators received foundational training in evidence-based psychotherapy theories and counseling skills (e.g., group facilitation, active listening, and basic therapeutic techniques) as part of their graduate curriculum. To prepare for this role, curriculum plans for each session were developed by program faculty and collaboratively refined with the trainee clinicians. Each session followed a structured guide that specified overall session learning objectives, goal of each session block, key content, and skill building activities to promote consistency across facilitators. Facilitators met with program faculty prior to each session, during which session objectives, content, and facilitation strategies were reviewed, and potential challenges were discussed. These meetings also provided an opportunity to clarify roles and ensure consistency in delivery across sessions. During the intervention period, facilitators received ongoing supervision from program faculty, including debriefing after sessions to address questions, reflect on group dynamics, reinforce adherence to core curriculum, and choose a topic for the next session. This combination of structured session guides, pre-session planning, and ongoing supervision was used to support standardization and consistency across session topics and facilitators.

### Procedure

Sessions used in this analysis took place between June 7, 2024 and June 4, 2025. Participants were asked to complete an optional online anonymous pre-assessment survey at the beginning of the session, which aimed to capture perception of resources needed to satisfy basic psychological needs at work, perceived stress, participant demographics, and anonymous participant IDs. In the pre-assessment, trainees were prompted to read an information sheet detailing the study, its procedures, their voluntary participation, and potential risks and benefits of participating. They were then prompted to provide consent to participate in the study. If consent was not given, they were automatically exited from the survey platform. After the session was completed, participants were prompted to take an optional anonymous post-assessment survey that retested participant perception of resources needed to satisfy basic psychological needs at work and perceived stress, with additional evaluation questions related to their experience with the session. Pre-and post-assessment data were collected using an online survey created and administered via Qualtrics (Provo, UT).

### Pre-Post Measures and Evaluation

Perception of resources needed to satisfy basic psychological needs at work were measured using the abbreviated 12-item Basic Psychological Need Satisfaction at Work (BPNS-W) scale, measured on a 5-point Likert scale (1 = Strongly disagree, 5 = Strongly agree) [49]. Items were adapted to capture trainee perception of the acquisition of resources needed to satisfy basic psychological needs (e.g., “I have the resources I need to express my ideas and opinions related to my work). Perceived stress was measured using a single-item measure [50]. To reflect participants’ immediate experience, the item was adapted to measure perceived stress at the time of the session instead of general stress experienced “these days.” BPNS-W and perceived stress were measured both pre- and post-session.

Single session evaluation measurement was captured using items from the Session Wants and Needs Outcome Measure (SWAN-OM) on a 5-point Likert scale (1 = Strongly disagree, 5 = Strongly agree). For the purposes of this study, post-assessment item measures were selected based on the overall intent of the sessions and corresponded to the reported most frequently selected items during single session evaluation (e.g., “I felt listened to.”) [51]. Two additional evaluation items were included in the post-assessment survey to capture participant perception of how helpful the session was and how likely the participant was to attend future sessions, measured on 5-point Likert scales (1 = Not helpful at all, 5 = Extremely helpful; 1 = Extremely unlikely, 5 = Extremely likely, respectively).

Demographic data were collected from participants in the pre-assessment and included trainee role, tenure in program, age, and race. Trainee role was collected using a closed-ended categorical variable with options including Graduate Student (Master or PhD), Professional (MD, MD/PhD, PA, MSN, NP, DNP, DPT, OTD), Postdoctoral Fellow, Medical Resident/Fellow, Other (with text entry option), and Prefer not to answer. Gender, race, and identity were also collected as closed-ended categorical variables, with the option to select all that apply. Tenure in program and age were collected as continuous variables using a text entry option requiring the number to be provided in years.

### Data Analysis

Raw data from 15 individual sessions over the study period were exported from Qualtrics and analyzed using IBM SPSS Statistics, (Version 29; Armonk, NY) and R (version 4.5.1; Vienna, Austria). Initial data cleaning and descriptive analyses were performed in SPSS. Consistent with the study’s design, each session was conceptualized and evaluated as an independent intervention exposure. Although some participants attended more than one session over the study period, pre- and post-session assessments were completed in relation to a specific session only, and analyses were conducted at the session level rather than longitudinally across participants. To assess whether repeat attendance introduced bias or violated assumptions of independence, we conducted a sensitivity analysis excluding repeat participants. The pattern and interpretation of the results were unchanged in the subsample, indicating that findings were not driven by cumulative exposure or repeat exposure. Accordingly, analyses included the full sample of participants with matched pre-post survey data as each session was treated as independent (*n* = 60). Unpaired pre- and post-responses were not analyzed, as participant attendance varied and exposure to the intervention could not be confirmed or assumed for those with incomplete survey pairs.

Mean-level pre-post changes in basic need satisfaction, using the BPNS-W scale, and perceived stress were first examined using paired-samples *t*-tests. Descriptive statistics were calculated for the post-test evaluative questions (SWAN-OM and general evaluation). Scale structure and reliability were evaluated in R. Factor analysis supported removal of reverse-coded items from the 12-item BPNS-W scale, resulting total of in a 9 items. Internal consistency was acceptable (α = 0.71) to excellent (α = 0.95) across subscales at pre-, post-, and pooled levels.

Consistent with study hypotheses, directional (one-tailed) tests were specified to examine increases in need satisfaction and decreases in perceived stress. Separate two-wave latent change score (LCS) models were estimated for competence, relatedness, and autonomy BPNS-W subscales [52, 53]. LCS modeling is preferred over simple paired *t*-tests because LCS models estimate change at the construct level while accounting for measurement error, providing a stronger test of true change. In addition, LCS models also allow evaluation of measurement equivalence across waves (i.e., pre- and post-test), ensuring that observed differences reflect meaningful change rather than shifts in how participants interpret items [52]. Indicators were treated as continuous, and missing data were handled using full information likelihood (FIML) allowing all available observations to contribute to model estimation.

To more rigorously test change over time, LCS modeling allowed us to model change as a latent variable, which captures within-person change over time, aligning with the study’s focus on how individuals respond to the intervention rather than relying solely on aggregate mean differences [52, 53]. We fit separate two-wave LCS models (i.e., constant change model and proportional change model) for autonomy, competence, and relatedness with a) metric invariance (equal loadings pre/post), b) minimal scalar invariance (one anchored intercept pair per construct), and c) same-item residual covariances across waves. Briefly, in constant change models, change is represented as a latent difference score with a freely estimated mean (μΔ), reflecting the average amount of change from pre- to post-intervention that is assumed to be independent of individual baseline scores. In proportional change models, change is modeled as a function of baseline scores via a proportional change parameter (βΔ ← pre) such that the magnitude and direction of change depend on individual initial standing on the construct. This specification captures processes such as regression to the mean or diminishing returns, where individuals with higher baseline values may show smaller gains over time.

## Results

A total of 116 trainees participated in the pre-assessment, and 80 participated in the post-assessment. Of this sample, a total of 60 participants provided participant IDs to create matched pre-post pairs (51.7% pre- and 75% post-assessment completion rate). The average age of the participants was 31.1 ± 7.3, 55% identified as women, and 40% were in the second year of their training program. Additional demographic details are presented in Table 1.

**Table 1:** Sample characteristics (*n* = 60)

**Training Role [Freq(%)]**	
Graduate Student (Master or PhD)	44 (73.3)
Professional Student (MD, MD/PhD, PA, MSN, NP, DNP, or DPT)	6 (10.0)
Medical Resident/Fellow	10 (16.7)
**Year in Program [Freq(%)]**	
Year 1	10 (16.7)
Year 2	24 (40.0)
Year 3	16 (26.7)
Year 4	6 (10.0)
Year 5	4 (6.7)
**Age [Mean (SD) [Min, Max]]**	31.1 (7.3) [22, 55]
**Gender Identity [Freq(%)]**	
Man	19 (31.7)
Woman	33 (55.0)
Other	8 (13.3)
**Race [Freq(%)]**	
White or European	26 (43.3)
Asian or Asian American	15 (25.0)
Black or African American	7 (11.7)
Other	12 (20.0)
**Identity [Freq(%)]**	
Parent and/or Primary Caregiver	21 (35.0)
LGBTQIA +	9 (15.0)
Having a Documented or Diagnosed Disability	7 (11.7)
First-generation College Student	20 (33.3)
English as a Second Language	9 (15.0)
Person of Color	15 (25.0)
International Student	9 (15.0)

*Freq* Frequency, *%* Percentage, *SD* Standard Deviation.

Factor loadings for the BPNS-W scale were strong and stable across time (Autonomy: 0.73–0.80 (pre) and 0.85–0.94 (post); Competence: 0.56–0.79 (pre) and 0.73–0.86 (post); Relatedness: 0.83–0.96 (pre) and 0.94–0.97 (post), indicating that the scale reliably measured each construct at both pre- and post-test. Single-construct models showed acceptable fit (Table 2).

**Table 2:** Global fit of latent change score models for basic psychological needs

Construct	χ^2^	*df*	CFI	TLI	RMSEA (90% CI)	SRMR
Competence	7.056	6	0.992	0.980	0.054 (0.000, 0.183)	0.066
Relatedness	4.489	6	1.000	1.009	0.000 (0.000, 0.142)	0.026
Autonomy	11.424	6	0.974	0.936	0.123 (0.000, 0.230)	0.073

Fit indices for single-construct latent change score (constant-change LCS) models. Estimator = MLR; Full information maximum likelihood (FIML) was used for missingness; metric invariance assumed equal factor loadings with a single anchored intercept pair and same-item cross-wave residual variances. *χ*^*2*^ , chi-square; *df,* degrees of freedom, *CFI*, comparative fit index; *TLI*, Tucker-Lewis index; *RMSEA*, root mean square error of approximation; *SRMR*, standardized root mean square residual.

Paired, one-tailed *t*-tests examined mean-level changes in 3-item BPNS-W summed scores for each construct (Table 3). Autonomy exhibited a positive gain (*M*Δ = 0.20; *p* = 0.030). Competence and relatedness showed trivial mean changes that were not statistically significant. Participants further reported lower perceived stress at post-test (*M* = 2.73, *SD* = 1.09) than pre (*M* = 3.07, *SD* = 1.10), representing a mean change of 0.33 (*p* < 0.001), reflecting an overall mean reduction in stress among participants.

**Table 3:** Pre-post changes in basic psychological need satisfaction and perceived stress

Construct	*n*	Pre *M* (*SD*)	Post *M* (*SD*)	Mean gain	*t*(df)	*p* (one‑tailed)	*dz*
Competence	60	4.07 (0.66)	4.07 (0.78)	0.00	0.00 (59)	0.500	0.00
Relatedness	60	4.07 (0.95)	4.10 (0.97)	0.03	0.33 (59)	0.372	0.04
Autonomy	60	3.87 (0.82)	4.07 (0.76)	0.20	1.92 (59)	0.030	0.25
Perceived Stress	60	3.07 (1.10)	2.73 (1.09)	−0.34	3.25 (59)	< 0.001	0.42

Paired one-tailed t-tests were conducted based on *a priori* predictions of improvement in psychological needs and reduction in stress. *M,* mean; *SD,* standard deviation; *dz,* standardized mean gain (mean gain/SD of gain); *df,* degrees of freedom.

Table 4 shows LCS parameters for competence, relatedness, and autonomy across both constant-change and proportional change models. Across both model types, there was no evidence of significant mean-level change from pre- to post- intervention. Although the direction of change was positive for autonomy and competence and slightly negative for relatedness, none of the LCS means (μΔ) reached statistical significance.

**Table 4:** Changes in basic psychological need satisfaction from pre- to post-session (*n* = 60)

**Panel A. Average Change from Pre- to Post-Session (Constant Change LCS)**							
**Construct**	**Average change (SE)**	***z***	***p***	**Individual differences in change (SE)**	***p***	**Baseline-change association (SE)**	***p***
Competence	0.08 (0.13)	0.65	0.26	0.53 (0.22)	0.018	− 0.15 (0.10)	0.112
Relatedness	− 0.02 (0.10)	− 0.16	0.57	0.35 (0.17)	0.041	− 0.15 (0.08)	0.049
Autonomy	0.12 (0.11)	1.03	0.15	0.45 (0.25)	0.069	− 0.23 (0.16)	0.137
**Panel B. Predicting Amount of Change Based on Starting Points (Proportional Change LCS)**							
**Construct**	**Baseline predicting change (SE)**	***p***	**Individual differences in change (SE)**	***p***	**Average change (SE)**	***z***	***p***
Competence	− 0.48 (0.23)	0.039	0.45 (0.16)	0.005	0.08 (0.10)	0.75	0.23
Relatedness	− 0.18 (0.09)	0.039	0.32 (0.15)	0.036	− 0.02 (0.10)	− 0.18	0.57
Autonomy	− 0.55 (0.23)	0.014	0.32 (0.14)	0.023	0.11 (0.09)	1.18	0.12

Panel A reports the average amount of change from pre- to post-session and whether participants differed from one another in how much they changed. Panel B reports whether participants’ starting levels predicted how much they changed. Average change tests use directional one-tailed *p* values; all other tests use two-tailed *p* values. *SE*, robust standard error.

However, significant variability in change was observed across constructs. In the constant-change models (Table 4, Panel A), variance in change scores [*p* Var(Δ)] was significant for competence (*p* = 0.018) and relatedness (*p* = 0.041), and marginal for autonomy (*p* = 0.069), indicating meaningful individual differences in how participants responded to the intervention. Further, the covariance between baseline scores and change in the constant change models was negative across constructs and reached statistical significance for relatedness (*p* = 0.049). This suggests that participants with lower initial relatedness tended to show greater increases over time.

Results from the proportional-change models further support this interpretation (Table 4, Panel B). A similar pattern in change variance was observed, wherein change variance remained significant, reinforcing meaningful between-person variability for all three constructs (competence, *p* = 0.005; relatedness, *p* = 0.041; autonomy, *p* = 0.023). The proportional change parameter (βΔ ← pre) was negative and statistically significant for competence (β = − 0.48; *p* = 0.039), relatedness (− 0.18; *p* = 0.039), and autonomy (β = − 0.55; *p* = 0.014). Thus, participants who started with lower scores experienced the largest gains, which were statistically significant across all three constructs from pre- to post-session.

Responses to post-test evaluative measures reflected good acceptability and self-reported improvement (Table 5). Across SWAN-OM items, “I felt listened to” was the most highly rated item (*M* = 4.22, *SD* = 0.83), followed by “I have found some ways to help myself” (*M* = 4.12, *SD* = 0.79), “I was able to explore how I feel” (*M* = 4.07, *SD* = 0.89), “I have identified some ways to help me worry less” (*M* = 4.02, *SD* = 0.88), “I was able to start feeling better” (*M* = 3.98, *SD* = 0.71), and “I better understand my feelings and/or behaviors” (*M* = 3.78, *SD* = 0.93). General evaluation items also reflected favorable impressions, as participants rated the sessions as helpful (*M* = 3.76, *SD* = 1.1), and indicated a likelihood of attending a future session (*M* = 4.03, *SD* = 1.1).

**Table 5:** Session evaluation (*n* = 59)

Session Wants and Needs Outcome Measure (*n*=59)	Mean	*SD*
1. I was able to start feeling better	3.98	(0.707)
2. I have identified some ways to help myself	4.12	(0.790)
3. I better understand my feelings and/or behaviors	3.78	(0.930)
4. I felt listened to	4.22	(0.832)
5. I have identified some ways to help me worry less	4.02	(0.881)
6. I was able to explore how I feel	4.07	(0.888)
General Evaluation (*n*=59)		
7. How helpful was today’s session?	3.76	(1.056)
8. How likely are you to come to another session in the future?	4.03	(1.082)

*SD,* Standard Deviation

Likert anchor statements for items 1–6 (1 = Strongly disagree, 5 = Strongly agree), 7 (1 = Not at all helpful, 5 = Extremely helpful), 8 (1 = Extremely unlikely, 5 = Extremely likely).

## Discussion

The current study describes a pilot evaluation of a novel intervention to reduce perceived stress and strengthen resources in support of basic psychological need satisfaction in health sciences trainees. Theoretically grounded in Self-Determination Theory, this effort sought to improve trainee perception of resource acquisition to assist in basic psychological need satisfaction with the goal of decreasing stress through participation in a peer-led intervention. Changes in basic psychological need satisfaction and perceived stress among health sciences trainees were evaluated, as well as trainees’ subjective evaluations of the intervention experience.

Mean-level pre- and post-intervention trainee self-reported BPNS-W scores improved in the domain of autonomy, suggesting that trainees may have particularly benefited from the skill-building component of the session, grounded in the SDT concept of autonomy. This supports the larger body of research calling for autonomy support for health sciences trainees within their training environments [9, 54, 55]. Studies show that these trainees often experience tightly structured schedules, limited control over their responsibilities, and hierarchical supervisory relationships that constrain opportunities for self-directed decision-making. These conditions contribute to a pervasive sense of restricted agency and have been linked to burnout, stress, and reduced intrinsic motivation [9, 56]. While causal inference is limited, the observed improvement in autonomy may reflect the targeted nature of the skill-building component, which aimed to support a sense of personal control and provide trainees with tools for practical application in their contexts. Together, these findings reinforce autonomy as a potentially malleable target for wellbeing-oriented interventions in health sciences education.

Significant mean-level changes were not observed in competence and relatedness from pre- to post-assessment. For competence, this may be attributed at least in part to a temporal lag between learning opportunities and the subjective experience of competence, which typically consolidates after individuals have had time to enact new behaviors. This is supported by the fact that competence is grounded in performance and mastery, which require application and practice rather than immediate results [57]. This is especially salient as the BPNS-W items measured satisfaction relative to gaining resources that aid in the reception of feedback, acquisition of new skills, and development of a sense of accomplishment. Similarly, the BPNS-W relatedness items asked participants about resource gains relative to their relationships with colleagues in their day-to-day work settings. It is possible that relational gains were instead experienced within the session itself through shared reflection and peer support but did not translate immediately to unique individual work environments. Future studies should consider assessing within-session relational dynamics and incorporate longitudinal follow-up to better capture delayed or context-dependent effects.

Despite modest group-level changes in BPNS-W, proportional-change LCS modeling revealed that participants with lower baseline levels exhibited statistically significant improvements across all basic psychological need domains. This suggests that participants with lower baseline scores experienced the greatest gains from the intervention, and that the intervention was most impactful for those with lower levels of basic psychological need satisfaction. This pattern aligns with the aggregate mean scores for competence and relatedness, which were relatively high at the time of the pre-assessment, suggesting that some participants may have had limited room for measurable improvement. Importantly, those with lower baseline need satisfaction my struggle more in their training programs and thus be more susceptible to poor mental health and academic outcomes. Considering this, the intervention described in this study could provide these vulnerable trainees with upstream support and timely assistance to address these adverse experiences and promote positive outcomes by offering a safe space with practical tools and resources.

Participants reported a significant reduction in perceived stress from pre- to post-session. Given the high burden of stress and burnout in the health sciences, this study offers promising evidence that even a brief intervention can meaningfully impact the wellbeing of trainees. This study suggests that short-duration interventions that provide space for reflection, validation, and emotional processing may help interrupt these stress cycles. The reduction in stress observed here may reflect the sessions’ emphasis on supportive listening and exploration of feelings and behaviors, elements that participants highly rated in the post-session evaluation. This highlights the value of integrating brief, accessible support opportunities into training programs to address acute stress and promote psychological recovery during periods of high demand.

Practically, the overall evaluation of the intervention was positive. The pattern of post-intervention ratings suggest that the intervention’s perceived value was largely in relational and agency-oriented processes. Feeling listened to was the most highly endorsed item, consistent with literature highlighting the importance of interpersonal validation and psychological safety in health sciences training environments [58, 59]. Extant literature has further shown that perceived support and feeling heard are associated with improved wellbeing, adaptation, and help-seeking among individuals [60]. Similarly, strong endorsement of identifying ways to help oneself aligns with evidence that enhancing self-efficacy is particularly salient for trainees [61–63] and a common mechanism in skills-based, preventative interventions [63–65]. The comparatively lower endorsement of items related to emotional understanding and symptomatic improvement may reflect the brief nature of the intervention, as deeper insight and sustained symptom change typically emerge over repeated engagement.

The results of this study should be interpreted within the scope of its limitations. This study was conducted at a single institution, and the sample size was modest (*n* = 60) and was largely composed of biomedical graduate trainees, potentially limiting its generalizability. Notably, 27% of the sample was heterogeneous health professions trainees, whose educational structures and expectations differ from those of biomedical graduate trainees. Sessions were designed to be broadly applicable across training contexts and the discussion-based format allowed participants to raise context-specific topics and challenges. Nevertheless, the findings, as well as variation across disciplines, may not generalize across diverse training contexts. While demographic characteristics of the participating trainees were captured and described in aggregate, the sample size did not support subgroup analyses. Future studies should examine how experiences and outcomes may vary across different trainee and demographic groups. Participants in this study may reflect a selection bias, such that trainees already both experiencing stress and interested in finding resources for its resolution would self-select for attendance. Some participants attended more than one session, so a sensitivity analysis was conducted that eliminated the repeat participants from the sample in order to assess their impact on the results of the study. Re-estimating the single-construct LCS models of only first-time participants did not alter the substantive conclusions relative to the full sample. Each session was treated as an independent intervention exposure, and the pre- and post-assessments were completed in relation to a specific session only. Given that each session covered a different topic, it is likely that new and different benefits were gained at each session. Convenience sampling was used to recruit study participants, which introduces the potential for selection bias. Future work should engage in more intentional, rather than convenience, sampling to better depict the universal benefit of this intervention for trainees. Further analyses with more rigorous design, such as randomized longitudinal approaches, are needed to elucidate causal inference and to examine the durability of intervention effects over time, as well as potential mechanisms of change. Such an analysis would also benefit from efforts to clarify which portions of the intervention are most valuable to trainees. Although a formal dismantling trial may not be realistic for this intervention model, post-evaluation questionnaires could aim to elucidate some of these uncertainties in future trials. Additionally, given that the intervention leaders were also trainees, there may be value to future trials in also assessing potential benefits to interventionists.

## Conclusion

This theory-driven, feasibility-oriented trial of a peer-led intervention among health sciences trainees provides preliminary evidence of meaningful effects, including significant reductions in perceived stress and improvements in at least one domain of basic psychological need satisfaction. Importantly, statistically significant improvements across all three domains were observed in participants with lower baseline scores. This suggests that this intervention may be particularly valuable for trainees who enter with unmet basic psychological need satisfaction and may be most vulnerable to the demands of health sciences training. Importantly, this intervention was positively received by participants and proved feasible to implement with minimal funding and administrative infrastructure, leveraging trainee clinicians as the primary interventionalists. Taken together, these findings suggest that this intervention design holds promise for supporting trainee wellbeing. Further trials are needed to confirm these findings, examine mechanisms of change, and evaluate potential for boarder implementation.

## Data Availability

The data presented in this study are not publicly available due to the sensitive nature of its contents. However, de-identified data are available upon reasonable request to the corresponding author.
